# The emerging role of SPOP protein in tumorigenesis and cancer therapy

**DOI:** 10.1186/s12943-019-1124-x

**Published:** 2020-01-04

**Authors:** Yizuo Song, Yichi Xu, Chunyu Pan, Linzhi Yan, Zhi-wei Wang, Xueqiong Zhu

**Affiliations:** 1 Department of Obstetrics and Gynecology, The Second Affiliated Hospital of Wenzhou Medical University, No. 109 Xueyuan Xi Road, Wenzhou, 325027 Zhejiang China; 2 Center of Scientific Research, The Second Affiliated Hospital of Wenzhou Medical University, No. 109 Xueyuan Xi Road, Wenzhou, 325027 Zhejiang China; 3 Department of Pathology, Beth Israel Deaconess Medical Center, Harvard Medical School, Boston, MA USA

**Keywords:** SPOP, Oncoprotein, Tumor suppressor, Cancer, Therapy

## Abstract

The nuclear speckle-type pox virus and zinc finger (POZ) protein (SPOP), a representative substrate-recognition subunit of the cullin-RING E3 ligase, has been characterized to play a dual role in tumorigenesis and cancer progression. Numerous studies have determined that SPOP suppresses tumorigenesis in a variety of human malignancies such as prostate, lung, colon, gastric, and liver cancers. However, several studies revealed that SPOP exhibited oncogenic function in kidney cancer, suggesting that SPOP could exert its biological function in a cancer type-specific manner. The role of SPOP in thyroid, cervical, ovarian, bone and neurologic cancers has yet to be determined. In this review article, we describe the structure and regulation of SPOP in human cancer. Moreover, we highlight the critical role of SPOP in tumorigenesis based on three major categories: physiological evidence (animal models), pathological evidence (human cancer specimens) and biochemical evidence (downstream ubiquitin substrates). Furthermore, we note that SPOP could be a promising therapeutic target for cancer treatment.

## Introduction

**Fig. 1:**
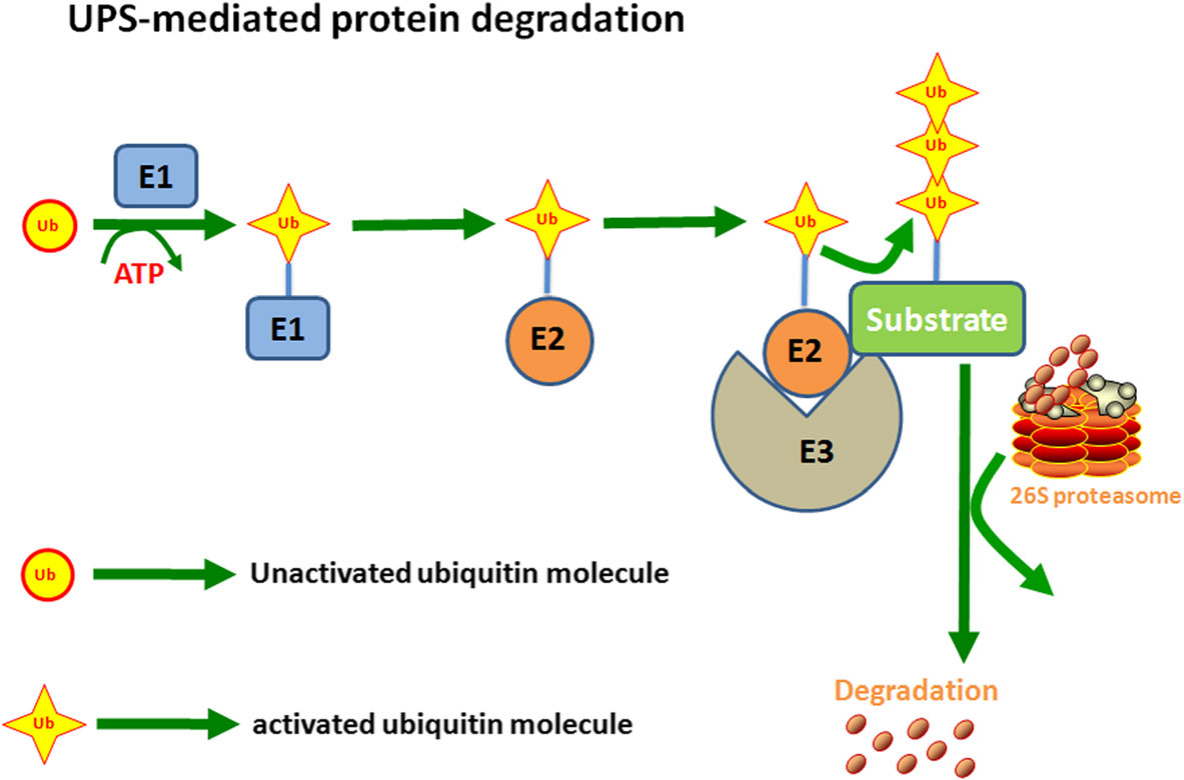
UPS-mediated protein degradation. Ubiquitin is activated by the E1 enzyme in an ATP-dependent fashion. Then, the active form of ubiquitin is conjugated to the E2 enzyme, after which it is transferred to a specific target substrate depending on the E3 ligase involved. E3 ligases are crucial for determining the substrate specificity for degradation

Protein degradation is critical for maintaining cellular homeostasis, and abnormal accumulation of proteins may lead to various diseases including human cancers [1]. There are two major proteolytic pathways that are conserved in eukaryotes: lysosomal-mediated proteolysis and proteasome-mediated degradation [2]. By promoting protein ubiquitination and degradation, the ubiquitin proteasome system (UPS) is responsible for the destruction of approximately 80% of intracellular proteins, thereby regulating an array of biological processes including cell proliferation, apoptosis, invasion and metastasis [3–5]. It is well accepted that UPS-mediated protein degradation is composed of two discrete steps: (1) a substrate protein is labeled by a single ubiquitin protein (monoubiquitination) or multiple ubiquitin molecules (polyubiquitination); (2) the ubiquitinated substrate is subsequently degraded by the 26S proteasome complex [6]. Biochemically, the first step of protein ubiquitination is achieved through three unique and consecutive enzymatic reactions, which are catalyzed by a ubiquitin-activating E1 enzyme, a ubiquitin-conjugating E2 enzyme and a ubiquitin-protein E3 ligase [7]. Mechanistically, ubiquitin is activated by the E1 enzyme in an ATP-dependent fashion. Then, the active form of ubiquitin is conjugated to the E2 enzyme, after which it is transferred to the specific target substrate depending on the E3 ligase involved [7]. Hence, E3 ligases are crucial for determining the substrate specificity for degradation (Fig. 1) [8].

**Fig. 2:**
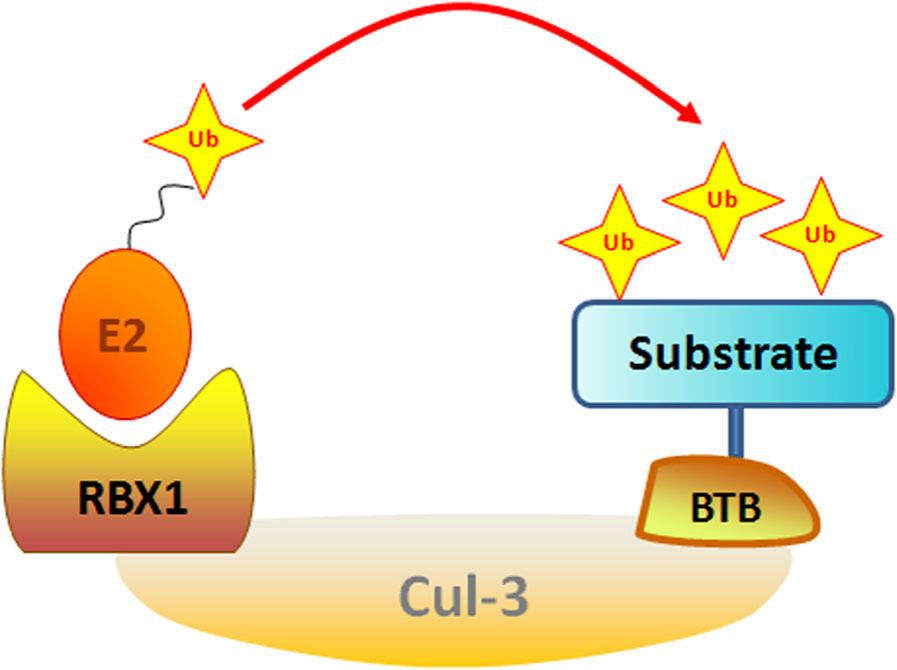
Cul-3 structure is illustrated. CRL3 contains three primary components including a Cul-3 protein as the scaffold protein, a RING-box protein (RBX1) that is essential for recruiting the E2 enzyme, and a Bric-a-brac-Tramtrack/Broad (BTB) protein; the BTB protein serves as both a substrate-recognition subunit (SRS) and the adaptor protein for substrate binding within this complex

The human genome encodes more than 600 putative E3 ligases [9], among which the cullin-RING E3 ligase (CRL) complex family is the largest and consists of eight members including CRL1, CRL2, CRL3, CRL4A, CRL4B, CRL5, CRL7 and CRL9 [10, 11]. Generally, the CRL E3 ligases are composed of a cullin (Cul) protein as the scaffold protein, a RING-box protein (RBX1 or RBX2) that is essential for recruiting the E2 enzyme, a substrate-recognition subunit (SRS) and an adaptor protein (SKP1, elongin B/C or DDB1) that links the SRS to the complex [12]. Interestingly, CRL3 contains only three primary components including the Cul-3 protein, the RBX1 protein and a Bric-a-brac-Tramtrack/Broad (BTB) protein, and the BTB protein serves as both the SRS and the adaptor protein for substrate binding within this complex (Fig. 2) [13, 14]. The nuclear speckle-type pox virus and zinc finger protein (SPOP), a representative SRS of CRL3, has been greatly explored for its dual functions in tumorigenesis [15, 16]. Therefore, in this review, we will mainly describe the structure and regulation of SPOP and discuss the role of SPOP in tumorigenesis on the basis of three major categories: physiological evidence (animal models), pathological evidence (human cancer specimens) and biochemical evidence (downstream ubiquitin substrates). We will also note that SPOP is a promising therapeutic target for cancer treatments.

### Structure of SPOP

**Fig. 3:**
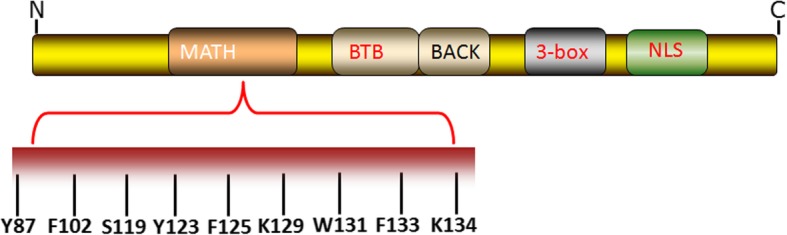
SPOP structure is illustrated. The SPOP protein comprises an N-terminal MATH domain, an internal BTB/POZ domain, a BACK domain, a 3-box domain and a C-terminal nuclear localization sequence (NLS). The MATH domain of SPOP selectively recognizes and recruits substrates and includes key amino acid residues such as Y87, F102, Y123, W131 and F133

SPOP was first discovered in 1997 by Nagai and his colleagues and harbors a typical POZ domain [17]. After that, accumulating evidence has elucidated the structure of SPOP. Structurally, the SPOP protein comprises an N-terminal MATH domain, an internal BTB/POZ domain, a BACK domain, a 3-box domain and a C-terminal nuclear localization sequence (NLS) (Fig. 3) [18, 19]. The MATH domain of SPOP plays a central role in selectively recognizing and recruiting substrates, and the majority of mutations are located in this domain [18]. This domain specifically recognized short linear motifs of substrates that serve as the specific SPOP-binding (SB) motifs. In turn, the substrate proteins require the existence of a prerequisite SB 5-residue motif φ-π-S-S/T-S/T (φ-nonpolar; π-polar amino acid), termed the SPOP-binding consensus (SBC) [18]. Moreover, the BTB domain is primarily involved in SPOP dimerization as well as Cul-3 binding. Specifically, an α3-β4 loop consisting of 10 amino acid residues in the BTB domain is crucial for this binding [20–22]. The BACK domain serves as a second place to mediate dimerization [19]. Furthermore, the 3-box domain, a pair of α-helices stretching beyond the BTB domain, has also been suggested to enhance the SPOP-Cul-3 interaction [23]. The dimerization interface in the BTB and BACK domains and the C-terminus work independently and form higher-order SPOP oligomers [24], which augments the E3 ligase activity by increasing the substrate avidity and the effective concentration of the E2 enzyme [23].

### Tumor suppressive role of SPOP in cancers

**Fig. 4:**
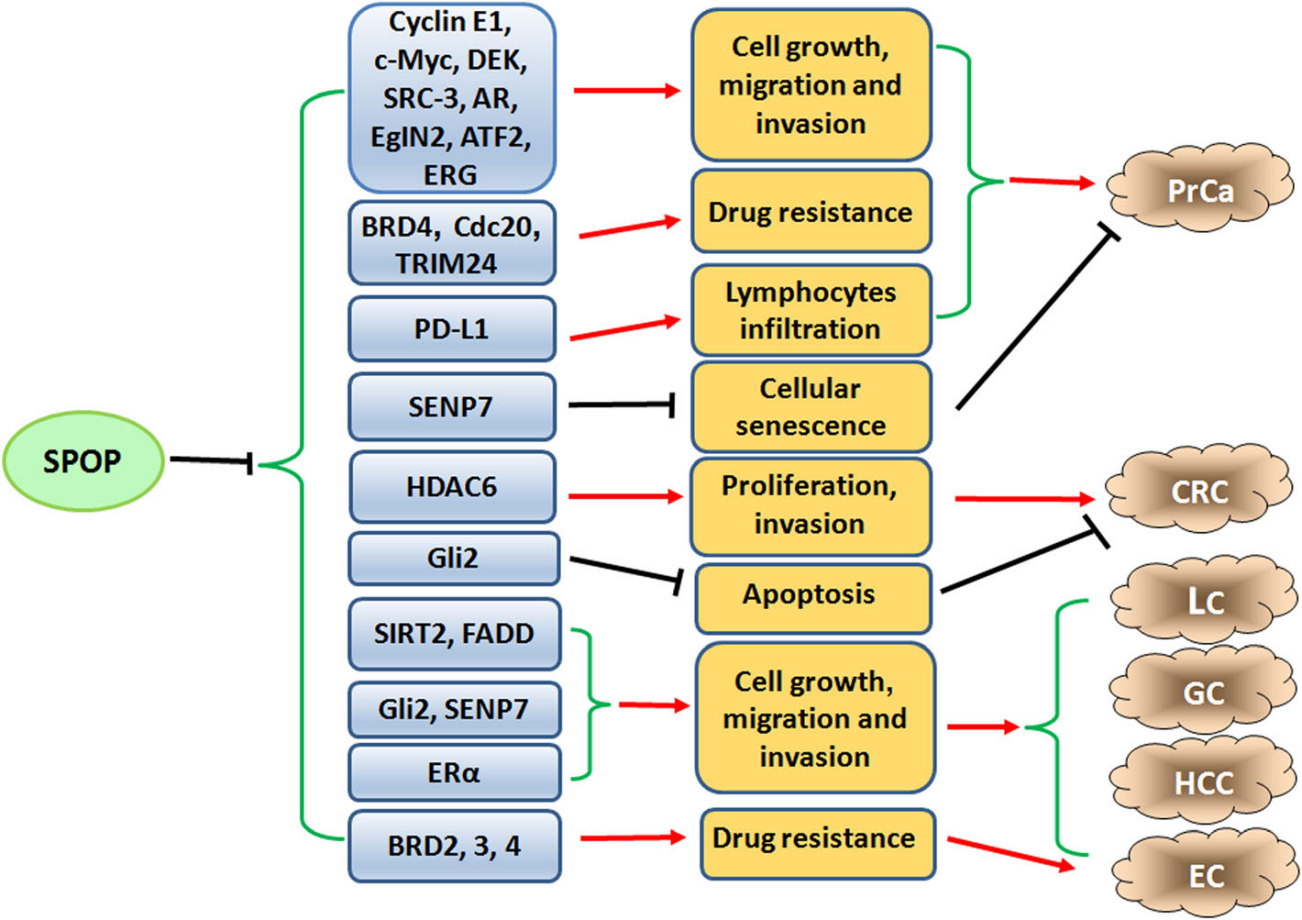
The tumor suppresssive role of SPOP in human cancers. SPOP suppresses tumorigenesis and progression via regulation of cell growth, apoptosis, migration, invasion and drug resistance by targeting its downstream substrates in several types of human malignancies, including prostate, lung, gastric, liver, colon and endometrial cancers

Multiple studies have determined that SPOP could suppress tumorigenesis in several types of human malignancies, including prostate, lung, gastric, liver, colon and endometrial cancers (Fig. 4, Table 1).

**Table 1:** SPOP plays a role in several types of human malignancies by targeting its downstream substrates

Cancer type	Physiological evidence (animal models)	Pathological evidence (human cancer specimens)	Biochemical evidence (downstream ubiquitin substrates)	Functions	Ref
BC	N/A	Mutation, reduced expression	c-Myc, SRC-3, PR	Suppression of tumorigenesis	[25–27]
CC	N/A	Increased expression, deletion mutant	None	Suppression of CC tumorigenesis	[28]
CRC	N/A	Mutation, reduced expression	Gli2, HDAC6	Inhibition of proliferation and migration, promotion of apoptosis and inhibition of tumorigenesis	[29–31]
EC	Conditional knockout SPOP–/–; infertility phenotype with reduction in PRs in the uterus	Mutation, reduced expression	BRD2, BRD3, BRD4, ERα	Inhibition of growth, suppression of EC tumorigenesis, enhanced sensitivity to BET inhibitors	[32–36]
GC	N/A	Decreased expression	Gli2	Inhibition of proliferation, migration and GC tumorigenesis	[37]
Glioma	N/A	SNPs, reduced expression	None	Suppression of glioma tumorigenesis	[38, 39]
HCC	Subcutaneous tumor model (SPOP-overexpression; tumor suppressive role)	Mutation, decreased expression	SENP7	Inhibition of migration and invasion, and HCC tumorigenesis	[40–42]
KC	Injection with transfected HEK293 cells (SPOP-cyto, tumorigenesis)	Overexpression	Daxx, DUSP7, Gli2, PTEN	Inhibition of cell apoptosis, promotion of cell proliferation and ccRCC tumorigenesis.	[43–46]
LC	N/A	Decreased expression	FADD, SIRT2	Inhibition of growth, suppression of LC tumorigenesis	[47, 48]
OC	N/A	Deletion mutant	None	Suppression of OC tumorigenesis	[49, 50]
OS	N/A	Decreased expression	None	Suppression of OS tumorigenesis	[51]
PrCa	Transgenic (expressing SPOP mutants); conditional knockout SPOP−/−); systemic knockout (embryonic lethal)	Mutation, decreased expression.	AR, ATF2, BRD4, cdc20, c-Myc, cyclin E1, DEK, EgIN2, ERG, PD-L1, SENP7, SRC-3, TRIM24.	Inhibition of PrCa development and progression	[52–76]
TC	N/A	Mutation	None	Suppression of TC tumorigenesis	[77–79]

#### Prostate cancer (PrCa)

PrCa is a common diagnosed cancer among men worldwide [80]. SPOP was reported as a frequently mutated gene in PrCa by Kan et al. in 2010 [52]. After that, whole-genome as well as exome sequencing analyses have revealed SPOP mutations in primary prostate neoplastic tumors but not in matched normal prostate tissues [53, 54]. SPOP mutations (such as Y87, F102, S119, Y123, F125, K129, W131, F133 and K134) often occur in the MATH domain of SPOP (Fig. 3) [54], and these mutations have been identified as an early event in the development of genomic instability and tumorigenesis in PrCa [55–58]. Notably, a number of animal models have been generated to explore the physiological role of SPOP in the development of PrCa and prostate tumorigenesis. For instance, homozygous deletion of *SPOP* (*SPOP*^*−/−*^) in zebrafish showed impaired brain, eye and body development that was largely rescued via microinjection of SPOP mRNA [58]. Furthermore, *SPOP*^*−/−*^ mice died between embryonic day 18.5 and postnatal day 1 [59]. Blattner et al. constructed a prostate-specific SPOP-F133 V mutation-carrying transgenic mouse and found that PrCa was developed in part due to the activation of the PI3K/mTOR and AR signaling pathways as well as the loss of *PTEN* [60]. Additionally, the PrCa-derived SPOP-F133 V mutation selectively damaged the homology-directed repair function mediated by wild-type SPOP (wt-SPOP) [58]. Clinical data showed that SPOP mutations and downregulation were detected in human PrCa tissues, and these mutations were also tightly correlated with a worse prognosis in patients with PrCa [61]. More importantly, extensive biochemical evidence has further indicated that SPOP functions as a tumor suppressor by promoting the degradation of oncogenic substrates in PrCa, including SRC3 [62], AR [63], TRIM24 [64], c-Myc [65], DEK [66], SENP7 [67], EglN2 [68], ATF2 [69], Cdc20 [70], ERG [71, 81], BRD4 [72–74], PD-L1 [75] and cyclin E1 [76]. Due to the many publications and space limitations, we will not describe the tumor suppressive role that SPOP plays by promoting the ubiquitination and degradation of its substrates in PrCa in detail. Therefore, we sincerely apologize to some researchers for not citing their important and meaningful papers.

#### Lung cancer (LC)

LC is one of the leading causes of cancer-related death in the world [82]. Downregulation of SPOP has been observed in non-small cell LC (NSCLC) tissues compared with normal tissues at both the transcriptional and translational levels [83]. Furthermore, the level of SPOP was confirmed to be associated with several clinicopathologic parameters, and a decrease in SPOP was considered a predictor of poor prognosis in patients with NSCLC, suggesting that SPOP could be a potential tumor suppressor in LC [83]. The sirtuin (SIRT) family of NAD-dependent protein lysine deacylases has been reported to participate in multiple biological processes such as transcription regulation, metabolism and DNA repair [84–86]. Notably, one group showed that SPOP promoted the proteasomal degradation of SIRT2 by binding to it, thus suppressing the growth of NSCLC cells [47]. Moreover, this ability was inhibited by mutation of SPOP in NSCLC cells. Furthermore, compared with the normal cells, NSCLC cell lines had elevated SIRT2 and reduced SPOP levels [47]. Fas-associated protein with death domain (FADD) is the key adaptor protein that transmits extrinsic apoptotic cell death signals by recruiting complexes of caspase 8 to death receptors [87, 88]. Emerging evidence has also shown that FADD expression is involved in tumorigenesis and cancer progression. For example, overexpression of FADD might serve as a biomarker in head and neck squamous cell carcinoma [89]. Furthermore, a high level of FADD protein has also been reported to be associated with poor outcome in LC, suggesting that it could become a potent prognostic biomarker in LC patients [48, 90]. Luo et al. found that SPOP directly bound to FADD and promoted its ubiquitination and degradation, blocking the development of NSCLC [48]. Therefore, SPOP exerts anticancer effects by targeting FADD in LC. Interestingly, an oncogenic role of SPOP in LC has also been indicated recently [91]. SPOP was found to be widely expressed in different LC cell lines. Conversely, knockdown of *SPOP* by shRNA in LC cells led to DNA damage repair defects, increased cell apoptosis and sensitization to irradiation under DNA damage conditions [91]. Therefore, in-depth investigation is essential to determine the role of SPOP in LC.

#### Gastric cancer (GC)

GC is one of the leading causes of cancer-related death worldwide and has a poor response to current chemotherapy [80]. The sonic hedgehog (Shh) signaling pathway is crucial for growth control and patterning during embryonic development and adult homeostasis [92]. Glioma-associated oncogenic (Gli) proteins are the main effectors of the Shh pathway, including Gli1, Gli2 and Gli3 [93, 94]. Among the three Gli proteins, Gli2 is the principal transcriptional activator that regulates Shh signaling in skin development and tumorigenesis. One study revealed that *Gli2*^*−/−*^ mice displayed hair morphogenesis defects similar to those of *Shh*^*−/−*^ mice [95]. Furthermore, a high level and increased activity of Gli2 in the epidermis is sufficient to promote the formation of basal cell carcinoma and maintain tumor growth [96, 97]. Moreover, upregulation of Gli2 protein was detected in GC specimens and Gli2 was also correlated with lymphovascular invasion in GC [98]. One group showed that the expression of SPOP was much lower in GC tissues than in adjacent normal tissues, while a high level of SPOP was negatively associated with poor clinicopathologic outcome [37]. Additionally, overexpression of SPOP significantly inhibited growth, metastasis, and colony formation in vitro. Mechanistically, using immunofluorescent staining, it was observed that elevation of SPOP accelerated the degradation of Gli2 without affecting its synthesis via intracellular interactions in GC cells [37]. However, whether SPOP functions as a tumor suppressor in GC should be further validated with more studies in the future.

#### Hepatocellular carcinoma (HCC)

HCC arises in patients as a consequence of long-standing preexisting liver illness, including viral hepatitis, alcohol abuse, and metabolic disease [99]. A previous study revealed using exome sequencing that SPOP was heterozygously mutated in one hepatoblastoma case and that it may normally play a tumor suppressive role in hepatoblastoma [40]. In addition, Huang et al. found a reduction in SPOP expression in HCC tissues and a low level of SPOP was associated with a high grade and intrahepatic metastasis in patients with HCC [41]. Moreover, in vitro experiments revealed that SPOP suppressed the growth and migration of HCC cells in part through blockade of ZEB2 expression [41]. Furthermore, one group also confirmed that the expression of SPOP was downregulated in HCC specimens and suggested SPOP as a predictor of poor prognosis for HCC patients [42]. Mechanistic studies implied that SPOP promoted the ubiquitin-mediated degradation and proteolysis of SENP7 by recognizing and binding to it, which eventually decreased the level of vimentin and attenuated the metastasis of HCC cells [42]. In addition, experiments on subcutaneous tumor mouse model also confirmed the inhibitory effect of SPOP on liver and lung metastases in HCC [42]. Overall, these studies indicate that SPOP might be a potential tumor suppressor in HCC.

#### Colorectal cancer (CRC)

CRC is the third most frequent cancer in the Western hemisphere and the incidence increases with increasing age [100]. Numerous studies have explored the role of SPOP in tumorigenesis and progression in CRC. Frequent downregulation of SPOP was detected in CRC tissues compared with paired adjacent normal tissues at both the mRNA and protein levels, and this downregulation was also significantly related to clinicopathologic parameters such as poor differentiation, distant metastasis, and high TNM stage [29]. Furthermore, a decrease in SPOP expression might serve as a potent predictive factor of poor prognosis for patients with CRC according to Kaplan-Meier survival analysis. In an in vitro study, overexpression of SPOP dramatically inhibited the proliferation and migration of CRC cells via upregulation of E-cadherin and downregulation of vimentin, MMP2, and MMP7, while this process was reversed by silencing of *SPOP* [29]. Moreover, one study also found that SPOP ablated MMP2 expression in CRC cells by suppressing the SP1 phosphorylation and nuclear translocation that was involved in the PI3K/Akt signaling pathway [101]. Histone deacetylase 6 (HDAC6) belongs to the HDACs family and is prominently involved in carcinogenesis and cancer progression [102, 103]. Numerous studies have found that high expression of HDAC6 exists in several human cancers and is associated with a significantly poor prognosis in diseases such as breast cancer [104] and PrCa [105]. HDAC6 contains two functional catalytic domains and deacetylates many nonhistone oncogenic proteins [103]. For example, HDAC6-mediated deacetylation leads to activation of HSP90 and promotes the binding of client proteins to HSP90 including AR, which enhances the activity of AR and suppresses its degradation [106, 107]. Additionally, the elevation of HDAC6 induced by P62 (also known as sequestosome-1, SQSTM1) promotes the epithelial-mesenchymal transition (EMT) process and impairs autophagic flux, facilitating the growth, migration and invasion of prostate cancer cells [108]. EMT is a reprogramming process in which epithelial cells take on mesenchymal phenotype after stimulation with EMT inducers [109]. The cells will lose the features of polarized immotile epithelial cells and obtain motile mesenchymal cell characteristics, leading to enhancement of migration and invasiveness [109]. Moreover, the expression of E-cadherin is decreased, while the levels of several mesenchymal markers including vimentin, Twist, ZEB1, ZEB2, and Slug are upregulated [109]. More importantly, multiple agents have been confirmed to exert their anticancer effects by inactivating and downregulating HDAC6 in PrCa cells [110–113]. It has been noted that SPOP specifically interacts with HDAC6 and promotes its polyubiquitination and degradation in human 293 T cells [30]. Notably, the growth and migration of *SPOP*-depleted colon cancer cells was partly reversed through knockdown of HDAC6 [30]. In line with the studies above describing the tumor suppressive function of SPOP in CRC, Zhi et al. showed that SPOP accelerated the ubiquitination and degradation of Gli2 by directly binding and interacting with it in CRC cells, enhancing the apoptotic signals of cells via a decrease in Bcl-2 and blocking the progression of CRC [31]. These results indicate that SPOP is a tumor suppressor in CRC, and it accomplishes this role by promoting the ubiquitination-mediated degradation of HDAC6 and Gli2.

#### Endometrial cancer (EC)

EC is the sixth most commonly diagnosed cancer in women worldwide [80], and multiple studies have been conducted to explore the function of SPOP in the tumorigenesis of EC. Hence, we will mainly discuss how SPOP plays a role in EC in this section. Recently, a *SPOP*^*−/−*^ mouse model was generated and exhibited an infertility phenotype with a reduction in progesterone receptors (PRs) in the uterus [114]. In addition, based on artificially induced decidualization and steroid hormone-processing mouse models, researchers have demonstrated that SPOP is required for embryonic implantation and for endometrial decidualization [115]. Collectively, these findings support a crucial role for SPOP in regulating normal uterine function. However, several genomic analyses have provided evidence that SPOP is frequently mutated in human EC [32–36], indicating that wild-type SPOP may act as a tumor suppressor in this disease. Zhang et al. discovered that estrogen receptor-α (ERα) is a specific substrate for SPOP, and ERα is also considered a main promoter of EC and facilitates the tumorigenesis of EC [36]. The SPOP protein accelerated the ubiquitin-mediated degradation of ERα by recognizing specific domains that contained abundant Ser/Thr (S/T)-rich degrons, and this action was reversed via the knockdown of *SPOP* by siRNAs in EC cells [36]. However, intriguingly, a recent study suggested that three BETs (BRD2, BRD3 and BRD4) were preferentially degraded by EC-related SPOP mutation and enhanced the sensitivity to BET inhibitors in EC cells [73]. Therefore, the definitive role of SPOP in the carcinogenesis of EC remains to be further elucidated in the future.

### Oncogenic role of SPOP in kidney cancer (KC)

**Fig. 5:**
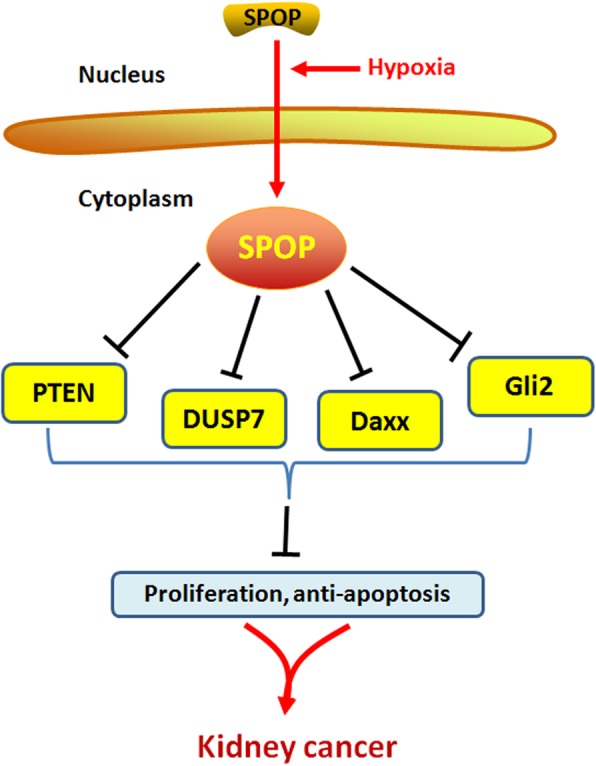
The oncogenic role of SPOP in kidney cancer. SPOP serves as an oncoprotein in kidney cancer by promotting the ubiquitination and degradation of PTEN, DUSP7, Daxx and Gli2, thus facilitating proliferation and inhibiting apoptosis in RCC cells

In contrast to the tumor-suppressive role of the SPOP protein in many human cancers described above, the oncogenic function of SPOP has been confirmed in KC. Studies have shown that SPOP is significantly upregulated in renal cell carcinoma (RCC) tissues at both the transcriptional and translational levels [43–45], and this upregulation was positively associated with cancer metastasis in patients with RCC [45]. In contrast, the malignant behaviors of RCC A498 and ACHN cells were reversed after SPOP knockdown using siRNA, as manifested by apoptosis induction, migration inhibition and increased sensitivity to sorafenib [116]. The SPOP protein has been identified as a nuclear protein in human normal embryonic 293 (HEK293) cells, whereas it has shown to be predominately transferred and accumulated in the cytoplasm of RCC cells under hypoxia [46]. Furthermore, Li et al. constructed stable polyclonal HEK293 cells transfected with cytoplasmic SPOP (cyto-SPOP) and found that 80% of nude mice injected with HEK293-cyto-SPOP developed tumor xenografts subcutaneously whereas no tumor growth was produced in the control groups [46]. Biochemically, cyto-SPOP promoted the ubiquitination and degradation of several tumor suppressors (including PTEN, DUSP7, Daxx and Gli2) in the cytoplasm, facilitating proliferation and inhibiting apoptosis in RCC cells [46]. Taken together, these findings suggest that SPOP plays an oncogenic role in KC cells via its cytoplasmic accumulation, resulting in degradation of tumor suppressive substrates of SPOP (Fig. 5, Table 1).

### SPOP functions to be determined in tumorigenesis

#### Breast cancer (BC)

**Fig. 6:**
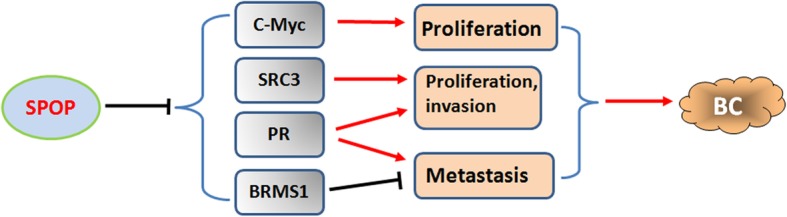
The dual roles of SPOP in breast cancer. SPOP might serve as a tumor suppressor or oncoprotein in breast cancer by regulating the degradation of its various substrates, but this role requires further validation

BC is one of the most common malignancies and is the second cause of death among women in the world [117]. One group reported that SPOP mediated the ubiquitination and degradation of c-Myc in triple-negative BC (TNBC) both in vitro and in vivo, while this process could be prevented by the lncRNA LINC01638, which interacted with c-Myc and subsequently promoted the expression of metadherin (MTDH) and Twist1 [25]. Moreover, Li et al. demonstrated that SPOP destabilized SRC-3 by hastening its polyubiquitination and proteasomal degradation in a phosphorylation-dependent manner in BC cells, thereby significantly reducing the levels of some molecules involved in SRC-3-mediated oncogenic signaling such as IGF-1 and MMP-2 [26]. In addition, knockdown of *SPOP* by shRNA promoted the proliferation and invasion of BC cells and elevated the cancer growth rate in a tumor xenograft mouse model, and these results were greatly influenced by upregulation of the SRC-3 oncoprotein [26]. For the majority of women with BC, tumor tissues show biological expression of receptors for estrogen and progesterone, hormones that are known to promote the growth and proliferation of cancer cells [118, 119]. PRs are member of the sex steroid receptor family, which modulates the function of sexual organs in a ligand-dependent manner, and PR has two coexpressed isoforms (PRA and PRB) [120]. There is compelling evidence suggesting that progesterone and PR play a crucial role in the development of BC [121, 122]. Gao et al. revealed that SPOP targeted PR for ubiquitin-mediated proteolysis and inhibited the PR transactivation induced by progesterone, S phase entry, and ERK1/2 activation, indicating that PR could be a bona fide substrate of SPOP in human BC [27]. Hence, these studies recommend that SPOP serves as a tumor suppressor by regulating the degradation of its substrates in BC. Breast cancer metastasis suppressor 1 (BRMS1) is a member of a subclass of the metastasis suppressor family, and its repressive effects on distant metastasis have been observed in several human cancers including BC [123, 124]. Intriguingly, one group suggested that BRMS1 may be a promising substrate that could be ubiquitinated and degraded by interacting with SPOP in BC cells [125]. Furthermore, deletion of *SPOP* dramatically promoted the expression of BRMS1 and decreased the level of OPN and uPA targeted and inhibited by BRMS1 in BC cells, suggesting that SPOP can also exert an oncogenic role in BC tumorigenesis and progression (Fig. 6) [125].

#### Cervical cancer (CC) and ovarian cancer (OC)

Although the prevalence of CC and OC survivors is on the rise due to improved outcomes after therapy, these diseases have also represented a serious threat to women’s health [126]. However, studies focusing on the role of SPOP in the tumorigenesis of CC and OC are still lacking. Only one group indicated that overexpression of the SPOP protein was essential for the apoptosis of HeLa cells, while this proapoptotic function was countered by ablation of *SPOP* [28]. Furthermore, one study used a tissue microarray to reveal a deletion mutant of *SPOP* in the majority of OC tissues that was not found in normal ovarian tissues, and this deletion mutant was correlated with high histological type and grade in OC [49]. Similarly, Jiang et al. identified several novel mutations in OC cell lines and tissues through wholeexome sequencing, including mutations in SPOP [50]. Thus, more studies should be carried out to clarify the function of SPOP in these two cancers and identify several potential substrates of SPOP during the carcinogenesis of CC and OC.

#### Thyroid cancer (TC)

TC is the most common endocrine malignancy worldwide [127], consisting of papillary (75–85%), follicular (10–20%), medullary (~ 5%) and anaplastic carcinomas (< 5%) [128]. Among the four types of TCs, only follicular thyroid carcinoma (FTC) has a benign counterpart named follicular thyroid adenoma (FTA). In fact, it has been widely accepted that FTA precedes FTC and has a favorable prognosis compared to FTC [129]. In this scenario, FTA originating from the thyroid follicle penetrates the tumor capsule and finally leads to the formation of FTC [130]. A few studies have investigated the genetic alterations responsible for the progression of FTA to FTC in recent years. Yoo et al. first identified the somatic mutation of SPOP-P94R in both minimally invasive FTC and FTA, and the mutations were confirmed by polymerase chain reaction (PCR) and Sanger sequencing assays [77]. Moreover, one group also found SPOP mutations in both FTC and FTA by conducting whole-exome sequencing and copy number profiling [78]. In addition, the results of evolutionary age analyses further showed that FTA genomes were as old as FTC genomes, implying the stability of the follicular thyroid tumor genomes during the transition from FTA to FTC [78]. Interestingly, one recent study found that adenomatoid nodules shared few overlapping gene mutations and expression patterns with coincidental papillary thyroid carcinoma (PTC) and the mutation of SPOP only existed in adenomatoid nodules [79]. Further studies should be performed in the future to explore several potential substrates of SPOP involved in the tumorigenesis.

#### Glioma and osteosarcoma (OS)

The function of SPOP has also been reported in glioma and osteosarcoma. Glioma is one of the most common primary brain tumors and has a high mortality worldwide [131]. A previous genome-wide linkage study revealed a series of single nucleotide polymorphisms (SNPs) in SPOP located on 17q12–21.32, and these SNPs may play an important role in gliomagenesis and cancer progression [38]. Furthermore, molecular studies found that SPOP was markedly downregulated in glioma samples compared to normal brain tissues, and low expression of SPOP displayed a potential for indicating poor prognosis in patients with glioma [39]. Conversely, overexpression of SPOP significantly suppressed glioma cell migration and invasion in vitro [39]. OS is a primary bone malignancy that predominantly occurs in children and adolescents [132]. Chen et al. showed that the expression of SPOP was decreased in both OS cell lines and clinical tissues [51]. Moreover, the migratory and invasive abilities of OS cells were dramatically enhanced after silencing of *SPOP*, whereas the anticancer effect was rescued by restoring the expression of SPOP, which negatively modulates the PI3K/Akt/NF-κB signaling pathway [51].

### Regulation of SPOP

The function of SPOP is regulated at different levels, including genetic alteration, and transcriptional, translational and post-transcriptional modifications (PTMs). Genetic alterations of SPOP, including mutation and aberrant expression, have been extensively observed in various human cancers and are discussed in the above text [133]. In addition, studies with regard to the PTMs of SPOP are still lacking. Therefore, in this section, we will mainly focus on transcriptional and translational regulation as well as other emerging mechanisms that modulate the biological functions of SPOP in physiological and tumorigenic processes. Reportedly, SPOP is a direct transcriptional target of hypoxic stress and hypoxia-inducible factors (HIFs) in clear cell renal carcinoma (ccRCC) [46]. In ccRCC cells, SPOP mRNA and protein levels are elevated under hypoxic conditions. Additionally, hypoxia is sufficient to induce the cytoplasmic accumulation of SPOP, which promotes the ubiquitin-mediated degradation of several tumor suppressors (Daxx, PTEN, DUSP7, and Gli2) and leads to tumorigenesis in kidney cancer [46]. Methylation, a common modification of DNA catalyzed by a family of DNA methyltransferase enzymes, directly regulates the transcription of SPOP by affecting its promoter [31, 134]. Zhi et al. indicated that hypermethylation of the specific CpG sites within the SPOP gene promoter region decreased the transcriptional activities of SPOP, thereby causing the progression and metastasis of CRC [31]. Several miRNAs have also been identified to be involved in the regulation of SPOP in human cancers. For example, miR-145 and miR-543 reduced endogenous SPOP levels in human CRC, BC, CC, HCC and GC cells by directly targeting a conserved putative binding site in the 3′-untranslated regions (3′-UTRs) of SPOP transcripts, promoting the invasion and migration of cancer cells [135, 136]. Additionally, one group independently showed that SPOP might be a new target of miR-372 and miR-373. Specifically, miR-372 and miR-373 enhanced the stemness of CRC cells and induced a poor differentiation status in CRC by inhibiting SPOP expression [137]. More recently, Zhang et al. also reported that miR-373 promoted the proliferation, invasion, and migration of oral squamous cell carcinoma (OSCC) cells by negatively regulating the expression of SPOP [138].

Liquid-liquid phase separation (LLPS) of proteins is an emerging and important mechanism for regulating the function of SPOP [139, 140]. During this process, multiple membraneless compartments are formed, through which enzymes and substrates are concentrated and protein degradation is enhanced [139]. Studies have shown that the SPOP protein exists in various nuclear bodies including nuclear speckles, promyelocytic leukemia (PML) bodies, DNA damage loci, and other substrate-containing bodies [17, 19]. Recently, Bouchard et al. found that localization of SPOP-substrate complexes in membraneless organelles triggered by LLPS was essential for SPOP-mediated ubiquitination and the subsequent degradation of specific substrates in cells [140]. Conversely, tumor-associated SPOP mutations disrupted LLPS and SPOP-substrate accumulation, thus inhibiting ubiquitin-dependent proteolysis of downstream proteins.

### SPOP as a therapeutic target

Since evidence has indicated oncogenic or suppressive roles of the SPOP protein depending on the specific cancer types, the SPOP protein could function as a novel therapeutic target for treating human cancers. Structurally, the SPOP protein can selectively bind to specific substrates by recognizing their SPOP-binding consensus (SBC) motif [18, 141]. On this basis, Quo et al. performed a computational screening and identified 109 small molecule inhibitors that disrupted the interactions between SPOP and its substrates [142]. Among these molecules, compound 6a showed the potential for competing with the puc_SBC1 peptide to bind to SPOP, while compound 6b was found to exert a strong inhibitory activity in disrupting the SPOP-PTEN and SPOP-DUSP7 interaction and blocking the downstream PTEN/Akt pathway in vitro, inhibiting the proliferation of A498 ccRCC cells [142]. Subsequent cellular thermal shift assay (CETSA) analysis further verified that compound 6b stabilized the substrates of SPOP in ccRCC cells [142]. Because the SPOP protein has context-dependent functions in different cancer types, future studies should focus on designing tissue- or cell-specific cancer drugs.

### Conclusions and perspectives

In conclusion, SPOP plays a dual role in the development and progression of human cancer by targeting its various substrates. Due to the oncogenic role of SPOP in kidney cancer, SPOP inhibitors are necessary for the suppression of SPOP to treat kidney cancer. To take advantage of the tumor suppressive role of SPOP in prostate cancer and other cancers, one alternative approach is to modulate the upstream effectors of SPOP, leading to the upregulation of SPOP. For example, downregulation of miR-145 and miR-543 could increase the SPOP level, and inhibit the migration and invasion of cancer cells [135, 136]. Downregulation of miR-372 and miR-373 could lead to upregulation of SPOP and subsequent inhibition of stemness in CRC cells [137].

Since most studies focus on the function and molecular mechanisms of SPOP in prostate cancer, the detailed role of SPOP in other tumors must be explored. Conditional engineered mouse models are important for determining the function of SPOP and the mechanism by which SPOP contributes to tumorigenesis and progression. Systematic approaches are needed to screen the substrates of SPOP in human cancer. It is also important to determine the reason why SPOP exhibits a dual role in certain tissues. Overall, further exploration is required to discover a rationale for designing therapeutic strategies using SPOP inhibitors or promoters for human cancer patients.

## Data Availability

Not applicable.

## Abbreviations

AR: Androgen receptor
ATF2: Activating Transcription Factor 2
BRMS1: Breast cancer metastasis suppressor 1
BTB: Bric-a-brac-Tramtrack/Broad
ccRCC: Clear cell renal carcinoma
cdc20: Cell division cycle 20
CETSA: Cellular thermal shift assay
CRC: Colorectal cancer
CRL: Cullin-RING E3 ligase
EC: Endometrial cancer
EMT: Epithelial-mesenchymal transition
ERα: Estrogen receptor-α
FADD: Fas-associated protein with death domain
FTA: Follicular thyroid adenoma
FTC: Follicular thyroid carcinoma
Gli: Glioma-associated oncogenic proteins
HCC: Hepatocellular carcinoma
HDAC6: Histone deacetylase 6
LIPS: Liquid-liquid phase separation
MMP: Matrix metalloproteinase
mTORC1: Mammalian target of rapamycin
NF-κB: Nuclear factor-kappa B
NLS: Nuclear localization sequence
NSCLC: Non-small cell lung cancer
OSCC: Oral squamous cell carcinoma
PCR: Polymerase chain reaction
PD-L1: Programmed death ligand 1
PI3K: Phosphatidylinositol 3-kinase
PML: Promyelocytic leukemia
PRs: Progesterone receptors
PTC: Papillary thyroid carcinoma
PTM: Post-transcriptional modification
RCC: Renal cell carcinoma
SBC: SPOP-binding consensus
SENP7: SUMO specific peptidase 7
SHH: Sonic hedgehog
SNPs: Single nucleotide polymorphisms
SPOP: Speckle-type pox virus and zinc finger protein
SRS: Substrate-recognition subunit
TNBC: Triple-negative breast cancer
TRIM24: Tripartite motif containing 24
uPA: Urokinase-type plasminogen activator
UPS: Ubiquitin proteasome system
UTR: Untranslated regions
